# Ruptured abdominal aortic aneurysm identified on point-of-care ultrasound in the emergency department

**DOI:** 10.1186/s12245-020-00279-9

**Published:** 2020-05-14

**Authors:** Omar Diaz, Wesley Eilbert

**Affiliations:** Department of Emergency Medicine, University of Illinois at Chicago, College of Medicine, Room 469, COME 1819 West Polk St, Chicago, IL 60612 USA

**Keywords:** Abdominal aortic aneurysm, Ruptured abdominal aortic aneurysm, Emergency ultrasound, Bedside ultrasound, Point-of-care ultrasound

## Abstract

**Background:**

Ruptured abdominal aortic aneurysm (AAA) is a highly lethal condition which requires rapid identification and treatment to improve the chance of survival. Computed tomography is the diagnostic modality of choice for ruptured AAA though it is time-consuming and often requires movement of the patient out of the emergency department (ED). Point-of-care ultrasound in the ED has excellent sensitivity and specificity for the detection of AAA, though less is known about its use to diagnose AAA rupture. We report a case of ruptured AAA identified on ultrasound performed at the bedside in the ED.

**Case presentation:**

A 77-year-old woman on warfarin with a known AAA presented to our ED with 2 days of epigastric abdominal pain. Point-of-care ultrasound revealed several findings suggestive of rupture of the AAA, which was confirmed on computed tomography. The patient was subsequently taken for emergent operative repair of the AAA and was later discharged from the hospital.

**Conclusions:**

Characteristics suggestive of AAA rupture may be seen on ultrasound. As ED physicians become more familiar with the use of point-of-care ultrasound in the evaluation of abdominal pain, identification of these characteristics may aid in the rapid diagnosis of AAA rupture.

## Background

Rupture of an abdominal aortic aneurysm (AAA) is often lethal, with a mortality of 85-90% [1]. Delays in the diagnosis and treatment are known to be a major contributor to the lethality of this condition [2]. A palpable abdominal mass may be present on physical exam, though this finding has a wide range of sensitivity from 29-76% [3]. With point-of-care ultrasound part of the core emergency medicine curriculum in the United States, emergency physicians have become quite adept at using this imaging modality to diagnose AAA [4]. However, less is known about the use of point-of-care ultrasound to diagnose AAA rupture. We report a case of a ruptured AAA identified by point-of-care ultrasound in the emergency department (ED).

## Case presentation

A 77-year-old woman presented to our ED complaining of non-radiating epigastric abdominal pain starting the previous day and becoming severe approximately 2 h before coming to the hospital. She had some associated non-bloody emesis. Her past medical history was significant for hypertension, peripheral vascular disease, coronary artery disease, chronic kidney disease, and chronic obstructive pulmonary disease with an extensive history of smoking. She was taking warfarin for protein S deficiency complicated by previous deep venous thromboses. She had a known infrarenal AAA measuring 4.4 cm in diameter seen on computed tomography (CT) 3 months earlier. The AAA had not changed in size over the previous 4 years. On arrival she appeared uncomfortable, though in no apparent distress. Her blood pressure was 142/113 mmHg, heart rate 60 bpm, respiratory rate 15 bpm, temperature 35.8 degrees Celsius, and pulse oximetry 97% on room air. She was noted to be tender to palpation in the epigastrium without guarding, and no masses were appreciated in her abdomen.

**Fig. 1:**
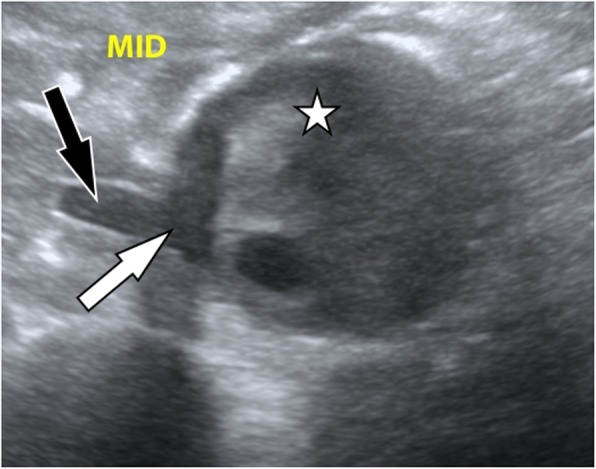
Ultrasonographic transverse view of the abdominal aortic aneurysm with an inhomogeneous appearance of the luminal thrombus (star), a focal disruption of the luminal thrombus and the outer wall of the aneurysm (white arrow), and a para-aortic hypoechoic area (black arrow)

**Fig. 2:**
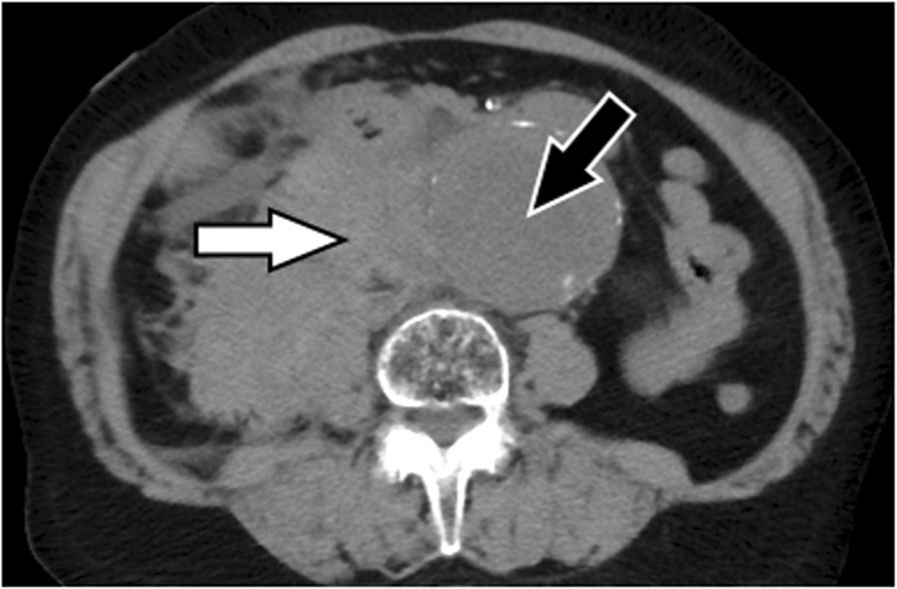
Noncontrast computed tomography of the abdominal aortic aneurysm (black arrow) with an adjacent subacute retroperitoneal hemorrhage (white arrow)

A point-of-care ultrasound was performed revealing an AAA measuring approximately 6 cm in diameter (Fig. 1). There was an inhomogeneous appearance of the luminal thrombus of the aneurysm. A hypoechoic area was identified which caused a discontinuity of the aneurysm’s luminal thrombus and outer wall and extended into the para-aortic space, suggestive of aneurysmal rupture. Laboratory values were significant for creatinine 2.0 mg/dL, international normalized ratio 1.4, and hemoglobin 10.6 g/L. A review of her previous laboratory values noted her hemoglobin had been 13.0 g/L approximately 2 months prior. CT of the abdomen and pelvis was performed confirming the presence of an infrarenal AAA measuring 6.4 cm in diameter (Fig. 2). The CT also revealed a large right retroperitoneal hematoma adjacent to the AAA with attenuation suggesting subacute hemorrhage.

A transfusion of 2 units of packed red blood cells was started in the ED, and the vascular surgery service was consulted. The patient was taken emergently to the operating room where the ruptured AAA was repaired using a polyester graft. After a difficult postoperative course, the patient was eventually discharged to a rehabilitation facility 5 weeks later.

## Discussion

The most commonly used definition of an AAA is a maximum infrarenal abdominal aortic diameter greater than 3 cm on ultrasound or CT imaging [5]. While rare under the age of 50, the prevalence of AAA over age 65 is 5-10% in men and 0.5-1.3% in women, with increasing prevalence with each decade of life [6, 7]. Risk factors for AAA include smoking, hypertension, white race, atherosclerotic disease, and a family history of the disorder [1]. The risk of rupture of AAAs less than 4 cm diameter is negligible; however, this risk increases exponentially after reaching a diameter of 5 cm to an annual rupture risk of 30-50% for those larger than 8 cm [8]. Unfortunately, most AAAs are asymptomatic until they rupture. It is estimated that ruptured AAA accounts for 1% of all deaths of men over age 65 and that 50% of patients with a ruptured AAA will die before reaching the hospital [9, 10].

Rapid identification and treatment of ruptured AAA is paramount to improving survival, though its diagnosis often remains elusive. It is estimated that only 30-50% of cases present with the classic triad of abdominal pain, hypotension, and pulsatile mass [3]. In 1992, Martin et al. found that 30% of cases were initially misdiagnosed, often as renal colic, diverticulitis, or gastrointestinal hemorrhage [11]. In 1994, Lederle et al. reported that the diagnosis of ruptured AAA was missed in 61% of cases presenting to internists and was only identified when hemodynamic compromise occurred [12].

CT is the diagnostic modality of choice for ruptured AAA, though point-of-care ultrasound is more readily available in most EDs. ED ultrasound screening for ruptured AAA has been found to reduce the time to diagnosis and improve patient outcomes [13]. ED point-of-care ultrasound has a sensitivity of 99% and specificity of 98% for the detection of AAA, though it is much less useful in identifying rupture. This is due to the fact that most (88%) AAAs rupture into the retroperitoneal space, where ultrasound visualization is limited [3]. In 2005, Catalano et al. described 8 sonographic findings of AAA rupture in 29 patients (Table 1) [14]. Four of these findings were present in our patient. Catalano et al. also described a contrast-enhanced bedside ultrasound using an intravenous microbubble contrast agent to improve the accuracy of diagnosing ruptured AAAs [15]. Some authors have advocated immediate operative repair of AAAs if rupture is identified on bedside ultrasound, especially in hypotensive patients [16]. This practice would bypass the time-consuming task of CT scanning.

**Table 1:** Sonographic findings with abdominal aortic aneurysm rupture

Deformation of aneurysmal shape	
Inhomogeneous appearance of the luminal thrombus	
Focal discontinuity of the thrombus layer	
A floating intraluminal thrombus layer	
Focal disruption of the outer aneurysmal wall	
A para-aortic hypoechoic area	
Retroperitoneal hematoma	
Hemoperitoneum	

Emergent operative repair has been the traditional treatment of ruptured AAAs, though endovascular repair has become another treatment option, even in unstable patients [17]. Regardless of the method of repair chosen, rapid identification of this highly lethal condition is of utmost importance. As emergency physicians become increasingly proficient with the use of bedside ultrasound, its role in the diagnosis of ruptured AAA will likely increase.

## Data Availability

Data sharing not applicable to this article as no data sets were generated or analyzed during the current study.
